# Amplicon sequencing of bacterial microbiota in abortion material from cattle

**DOI:** 10.1186/s13567-017-0470-1

**Published:** 2017-10-10

**Authors:** Sara Vidal, Kristel Kegler, Horst Posthaus, Vincent Perreten, Sabrina Rodriguez-Campos

**Affiliations:** 1 Institute of Veterinary Bacteriology, Vetsuisse Faculty, University of Bern, Laenggassstrasse 122, 3012 Bern, Switzerland; 2 Graduate School for Cellular and Biomedical Sciences, Theodor Kocher Institute, University of Bern, Freiestrasse 1, 3001 Bern, Switzerland; 3 Institute of Animal Pathology, Vetsuisse Faculty, University of Bern, Laenggassstrasse 122, 3012 Bern, Switzerland

## Abstract

Abortions in cattle have a significant economic impact on animal husbandry and require prompt diagnosis for surveillance of epizootic infectious agents. Since most abortions are not epizootic but sporadic with often undetected etiologies, this study examined the bacterial community present in the placenta (PL, *n* = 32) and fetal abomasal content (AC, *n* = 49) in 64 cases of bovine abortion by next generation sequencing (NGS) of the 16S rRNA gene. The PL and AC from three fetuses of dams that died from non-infectious reasons were included as controls. All samples were analyzed by bacterial culture, and 17 were examined by histopathology. We observed 922 OTUs overall and 267 taxa at the genus level. No detectable bacterial DNA was present in the control samples. The microbial profiles of the PL and AC differed significantly, both in their composition (PERMANOVA), species richness and Chao-1 (Mann–Whitney test). In both organs, *Pseudomonas* was the most abundant genus. The combination of NGS and culture identified opportunistic pathogens of interest in placentas with lesions, such as *Vibrio metschnikovii, Streptococcus uberis*, *Lactococcus lactis* and *Escherichia coli.* In placentas with lesions where culturing was unsuccessful, *Pseudomonas* and unidentified *Aeromonadaceae* were identified by NGS displaying high number of reads. Three cases with multiple possible etiologies and placentas presenting lesions were detected by NGS. Amplicon sequencing has the potential to uncover unknown etiological agents. These new insights on cattle abortion extend our focus to previously understudied opportunistic abortive bacteria.

## Introduction

Infectious abortion in ruminants is a problem in animal husbandry worldwide. Its importance is related not only to economic loss in animal production but also to infectious risks posed to humans and other animals [1, 2].

The most common bacterial abortive agents involved in ruminant abortion are *Brucella* spp., *Coxiella burnetii* and *Chlamydia abortus* [2–4]. While *Brucella* spp. and *C. burnetii* are typically involved in bovine fertility problems as abortion, stillbirth and weak offspring [5], chlamydial infections may cause a variety of syndromes including conjunctivitis, polyarthritis, encephalomyelitis, mastitis and other urogenital tract infections [2]. In humans, brucellosis, Q fever (*C. burnetii*) and chlamydiosis should be considered among the most common zoonotic diseases around the world [2, 5]. Moreover, there is increasing evidence supporting the implication in bovine abortion of other *Chlamydia*-related bacteria [2, 4, 6]. Other bacterial agents sporadically associated with bovine abortion that can also cause serious zoonotic diseases are *Salmonella* spp., *Campylobacter* spp., *Leptospira* spp. and *Listeria monocytogenes* [2, 5]. However, many of the bacterial causes of abortion involve opportunistic pathogens and often remain undetected. These organisms are common inhabitants in the host and environment and can occasionally enter the blood stream of the dam, subsequently infecting the placenta and producing sporadic abortion [7].

Laboratories conducting abortion diagnostic examinations should perform standard tests covering the major abortive infectious diseases; however, costs dictate that these tests are limited to the most common etiologies [8]. For example, first-line routine bacteriological abortion diagnostics in cattle in Switzerland only include serology and staining for *B. abortus* and *C. burnetii* (Swiss ordinance on epizootic diseases, article 129). In general, success rates for abortion diagnoses are low. Rates ranging from 23.3 to 45.5% were reported in the United States and a diagnostic rate of 22.5% in England, Wales and Scotland [9, 10]. While positive results for the before-mentioned diseases are notifiable and gathered in a national database, this does not imply causality of an abortion and, hence, no true diagnostic rates can be reported from Switzerland. In addition, problems such as inappropriate sample collection and submission, incomplete case history, environmental contamination and/or poor condition of the fetus may further hamper achieving an etiological diagnosis [7]. The placenta is considered the most useful sample; however, after abortion, the placenta is exposed to several environmental contaminants and detection of an agent in the placenta only does not imply that it actually passed on to the fetus. Thus, fetal abomasal fluid and organs are also required for culture and molecular detection [5].

Increasing knowledge of the bacteria that are involved in cattle abortion is crucial for optimizing the diagnostic approach and revealing emerging pathogens. Culture-independent DNA technology based on sequencing of the *rrs* gene encoding 16S rRNA has the potential to uncover both known and novel microorganisms [11]. Thus far, no study has focused on the bacterial microbiota present in bovine abortion material.

The objective of this study was to characterize the microbiota of samples from cattle abortions using high throughput sequencing of the V3–V5 region of the 16S rRNA gene to provide new insight into the bacteria that may play a role in bovine abortion.

## Materials and methods

### Sample collection

Samples from 64 bovine abortion cases from different cantons of Switzerland that were submitted for routine abortion investigation were collected from October 2012 to March 2014 [Bern (*n* = 40), Luzern (*n* = 6), Solothurn (*n* = 5), Aargau (*n* = 3), Vaud (*n* = 3), Basel-Land (*n* = 2), Fribourg (*n* = 2), Jura (*n* = 2) and Valais (*n* = 1)]. From the 64 cases of aborted cows, we analyzed 81 samples [32 samples of placenta (PL) and 49 of fetal abomasal content (AC)]. Samples of placenta and fetal organs were handled in the microbiology laboratory under the laminar flow hood. The fetal abomasal content was obtained by puncture with a sterile needle and syringe and was transferred to a sterile tube.

As negative controls we included healthy placentas and fetuses obtained from cows in calf submitted for necropsy to determine the cause of death of the dam by routine diagnostics. Sampling was done under aseptic conditions disinfecting the surface prior to puncture/incision with an antimicrobial solution (Neoform K Spray, Dr Weigert GmbH & Co. KG, Hamburg, Germany). The amniotic fluid was extracted by puncture with a sterile syringe and transferred to a sterile tube; the uterus was opened with a sterile scalpel and a cotyledon of the PL was transferred to a sterile container. The samples were relocated to the microbiology laboratory in an aseptic tray and processed as described above. The samples were used as negative controls after confirmation of the cause of death of the dam as non-infectious.

### DNA extraction from the PL, AC and amniotic fluid (AF)

For the extraction of the total genomic DNA, a piece of PL (approximately 2 cm) was cut and suspended in 5 mL of 0.85% NaCl in an IKA^®^ DT-20 tube [12]. For the extraction of the AC/AF DNA, 1 mL of content was used. The tissue and the content were homogenized twice for 30 s at 6000 rpm, using the IKA ULTRA-TURRAX^®^ tube drive (IKA^®^-Werke GmbH & Co. KG, Staufen, Germany). From the homogenate, 500 µL were used to make the DNA extraction using the PowerSoil^®^ DNA Isolation Kit (Mobio, Carlsbad, CA, USA). Fluorometric quantification was performed to test the DNA quantity using the Quantus™ Fluorometer (Promega, Dübendorf, Switzerland). The DNA extraction procedure was performed with two extraction control tubes containing only reagents.

### PCR, Illumina MiSeq sequencing and sequence data processing

A classic polymerase chain reaction (PCR) amplification of the 16S rRNA hypervariable V3-V5 region was performed to verify the presence of bacterial DNA before sequencing. The primers used were: 357F_hmp (5′-CCT ACG GGA GGC AGC AG-3′) and 929R_hmp (5′-CCG TCA ATT CMT TTR AGT-3′). PCR was performed at a final volume of 30 µL of reaction mixture containing: 1X PCR buffer, 2 mM MgCl2, 0.4 µM forward and reverse primer, 200 µM dNTPs, 0.25 µL of 5 U/µL thermostable DNA FIREPol^®^ Polymerase Solis BioDyne, 21.25 µL of sterile water and 2 µL of DNA solution. The following conditions were applied: 94 °C for 3 min, followed by 35 cycles of 95 °C for 30 s, 54 °C for 30 s, 72 °C for 45 s, and a final elongation step at 72 °C for 8 min. Amplification was carried out in a Biometra T professional gradient Thermocycler (Biometra GmbH, Göttingen, Germany) and the PCR products were analyzed by agarose gel electrophoresis with a 100-bp DNA ladder as a molecular weight marker (Promega AG, Dübendorf, Switzerland) to check the products for the expected size (572-bp). We included pure genomic DNA from *Escherichia coli* and water as positive and negative controls for the PCR, respectively. PCR-positive samples presenting a concentration of ≥ 10 ng/µL by the Quantus™ Fluorometer were considered suitable for sequencing. The two negative control tubes were included in the PCR.

To sequence the V3–V5 regions of the bacterial 16S rRNA gene, two-step PCR libraries using the primer pairs 357F_hmp and 929R_hmp were created. Subsequently, the Illumina MiSeq platform and a v3 600 cycle kit were used to sequence the PCR libraries. The produced paired-end reads that passed Illumina’s chastity filter were subjected to demultiplexing and trimming of Illumina adaptor residuals (no further refinement or selection). The read quality was checked with FastQC software (version 0.11.5) [13]. Locus specific V345 adaptors were trimmed from the sequencing reads with Cutadapt v1.9.2.dev0 [14]. Paired-end reads were discarded when the adaptor could not be trimmed. Trimmed forward and reverse reads of the paired-end reads were merged using a minimum overlap of 15 bases. Sequences were then quality filtered allowing a maximum of one expected error per merged read, and those containing ambiguous bases were discarded. The resulting data were clustered by USEARCH version 8.1.1861 [15] at a 97% identity level to form operational taxonomic units (OTUs) while discarding singletons and chimeras in the process [16]. OTUs were aligned against the core set of the Greengenes v13.8 [17] database, and taxonomy was predicted with a minimum confidence threshold of 0.7. Libraries, sequencing and generation of the OTU table were performed at Microsynth AG (Balgach, Switzerland). The core microbiome was calculated from the BIOM table to group the samples by organ (PL or AC). The bacterial distribution at the phylum, class, order, family and genus level was summarized and plotted using the script *summarize_taxa_through_plots.py* in QIIME 1.9.1 [18].

### Statistical analysis

The sequencing depth was normalized by sub-sampling the dataset randomly to 9000 reads per sample. The OTU dataset was normalized by log2-transformation. Paleontological Statistics (PAST; v3.12) software [19] was used for alpha-diversity analyses including observed species richness, the mean number of OTUs; Shannon Diversity Index, a measure of species that combines species abundance and evenness; and Chao-1, an estimation of true species diversity. Data ordination by principal component analysis (PCA) and assessment of differences between microbial profiles of the two groups by one-way PERMANOVA (Bray–Curtis similarity distance) was performed. The significant differences in alpha-diversity were calculated in both types of abortion samples, AC and PL using the Mann–Whitney U test in XLSTAT 2012 software (Addinsoft, Barcelona, Spain). The *p* values were corrected using Bonferroni correction. *p* < 0.05 were considered statistically significant.

### Isolation and identification of bacteria—broad spectrum culture

For the identification of culturable bacteria present in the abortion material (PL and AC) and in the healthy fetuses (PL, AC and AF), 100 µL from the homogenized samples were cultured in trypticase soy agar with 5% sheep blood, MacConkey agar and PALCAM Listeria agar for up to 48 h at 37 °C. Additionally, 1000 µL of the homogenate were enriched in Müller–Kauffmann Tetrathionate-Novobiocin broth for detection of *Salmonella* spp., for 24 h at 37 °C, and 100 µL were then plated onto Brilliant Green Agar and Salmonella Chromagar (Oxoid) and incubated at 37 °C for 24 h. For detection of *Campylobacter* spp., 1000 µL of the homogenate were enriched in Thomann Transport and Enrichment medium [20] for 48 h at 37 °C, and 100 µL were plated onto Skirrow agar (Oxoid) after incubation at 37 °C in a microaerophilic atmosphere for 5 days. The isolates were identified by matrix-assisted laser desorption/ionization time-of-flight mass spectroscopy (MALDI-TOF MS) (Biotyper 3.0, Bruker, Daltonics GmbH, Bremen, Germany) using the direct transfer protocol recommended by the manufacturer.

### Histopathology

To establish a correlation between the sequencing analysis and pathological changes, we evaluated a subset of 17 placentas histopathologically. Cases with positive results for parasitology (including *Toxoplasma gondii* and *Neospora caninum*), virology (including bovine viral diarrhea virus and Schmallenberg virus) and/or presence of intralesional fungal organisms were excluded to avoid misinterpretation of etiological causes. The parasitological and virological analyses were carried out upon senders’ request at the Institute of Parasitology, Vetsuisse Faculty, and the Institute of Virology and Immunology (University of Bern, Switzerland), respectively, and are not further discussed in the present study. Samples of placental cotyledons were fixed in 10% buffered formalin and routinely embedded in paraffin. Sections (4 μm) were mounted on Thermo Scientific™ SuperFrost Plus© (Braunschweig, Germany) slides and stained with hematoxylin and eosin (HE).

## Results

### 16S rRNA gene—PCR screening

We confirmed the presence of bacterial DNA in the 81 samples of abortion material. The healthy fetal samples were all negative; no bacterial DNA was amplified in the control samples including the two negative extraction control tubes.

### Sequencing overview

A total of 81 samples from 64 bovine abortion cases, were analyzed to investigate the composition of the bacterial microbiota. Samples were used to generate deep V3–V5 16S rRNA gene profiles. A total of 5 220 804 high-quality reads were obtained, with an average of 64 454.37 ± 33 447.726 sequences per sample. The overall number of OTUs detected was 992 based on a 97% nucleotide sequence identity between reads. The number of reads per sample ranged from 9 918 to 169 878 (median 61 745; mean 64 454; SD 33 448). After sub-sampling 9 000 reads/sample, 913 OTUs remained in the dataset that was used for further analysis.

### Microbial profile analysis

**Figure 1:**
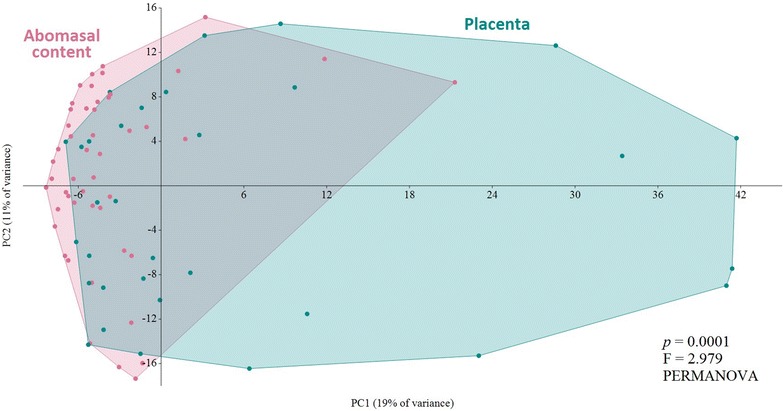
**Two-dimensional ordination of the microbial profiles of the abomasal content and placenta by principal component analysis (PCA).** Significant differences; *p* < 0.01, PERMANOVA.

**Figure 2:**
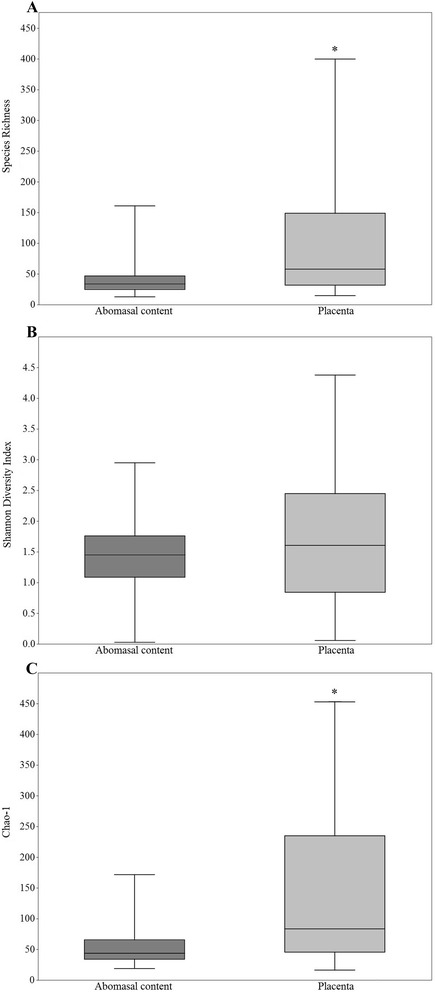
**Diversity analysis: microbial profiles of the abomasal content and placenta. A** Observed species richness; **B** Shannon Diversity Index, **C** Chao-1. *Significant differences; *p* < 0.01, Mann–Whitney test.

Principal component analysis showed significant clustering by organ (*p* = 0.0001, F = 2.979, PERMANOVA) (Figure 1). Microbial profiles from the PL showed significant higher values of actual species richness (number of OTUs) (Figure 2A) and the estimated species richness or Chao-1 (Figure 2C). The Shannon Diversity Index did not show significant differences (Figure 2B). On average, samples from the AC showed 42 ± 1 (SEM) OTUs, whereas samples from the PL showed 110 ± 1 (SEM) OTUs.

### Composition of the associated bacterial communities in the AC and PL

**Figure 3:**
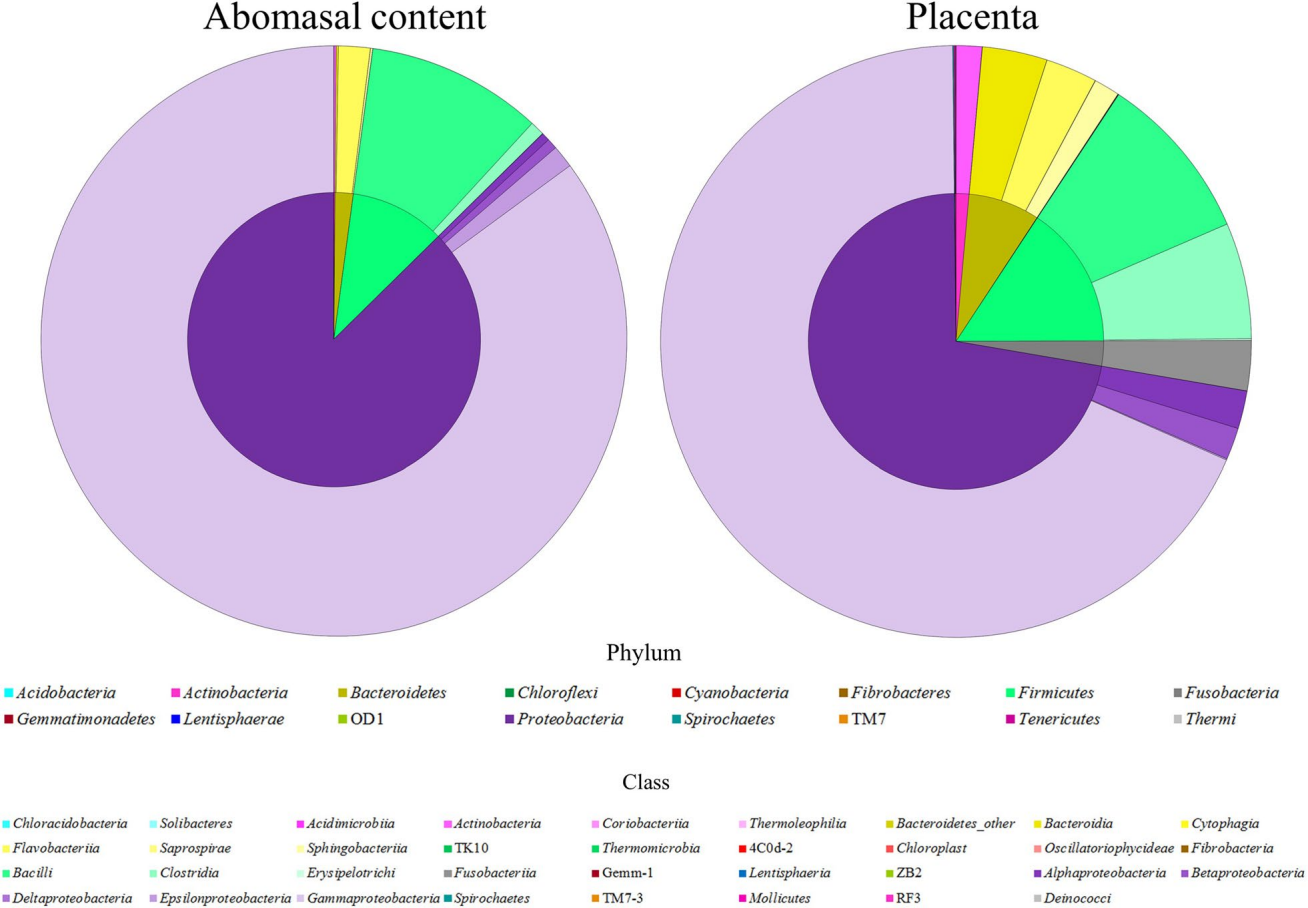
**Relative abundance of phyla (internal circle) and class (external circle) in the abomasal content and placenta.**

At the phylum and class level, 16 and 35 subcategories were identified in the abortion material samples, respectively (Figure 3). The number of phyla found in the PL and AC were 15 and 9, respectively, while the number of shared phyla was 9 (Table 1). The three predominant phyla were Proteobacteria (AC = 87.35%; PL = 72.13%), Firmicutes (AC = 10.51%; PL = 15.66%) and Bacteroidetes (AC = 1.99%; PL = 7.81%), accounting for 99.85% of the bacterial communities in the AC and 95.6% in the PL (Table 1). Only Proteobacteria and Firmicutes were present in all samples. Of the 35 class level-subcategories, 34 were found in the PL, whereas only 18 were present in the AC.

**Table 1:** **Phylum-level composition. Relative abundance of phyla in the abomasal content and placenta**

Phylum	Abomasal content (%)	Placenta (%)
Acidobacteria	–	0.004
Actinobacteria	0.13	1.42
Bacteroidetes	1.99	7.81
Chloroflexi	–	0.001
Cyanobacteria	0.01	0.03
Fibrobacteres	–	0.02
Firmicutes	10.51	15.66
Fusobacteria	0.01	2.74
Gemmatimonadetes	–	0.0003
Lentisphaerae	–	0.01
OD1	0.0005	–
Proteobacteria	87.35	72.13
Spirochaetes	0.003	0.07
TM7	–	0.0003
Tenericutes	0.00431	0.10
Thermi	–	0.01

**Figure 4:**
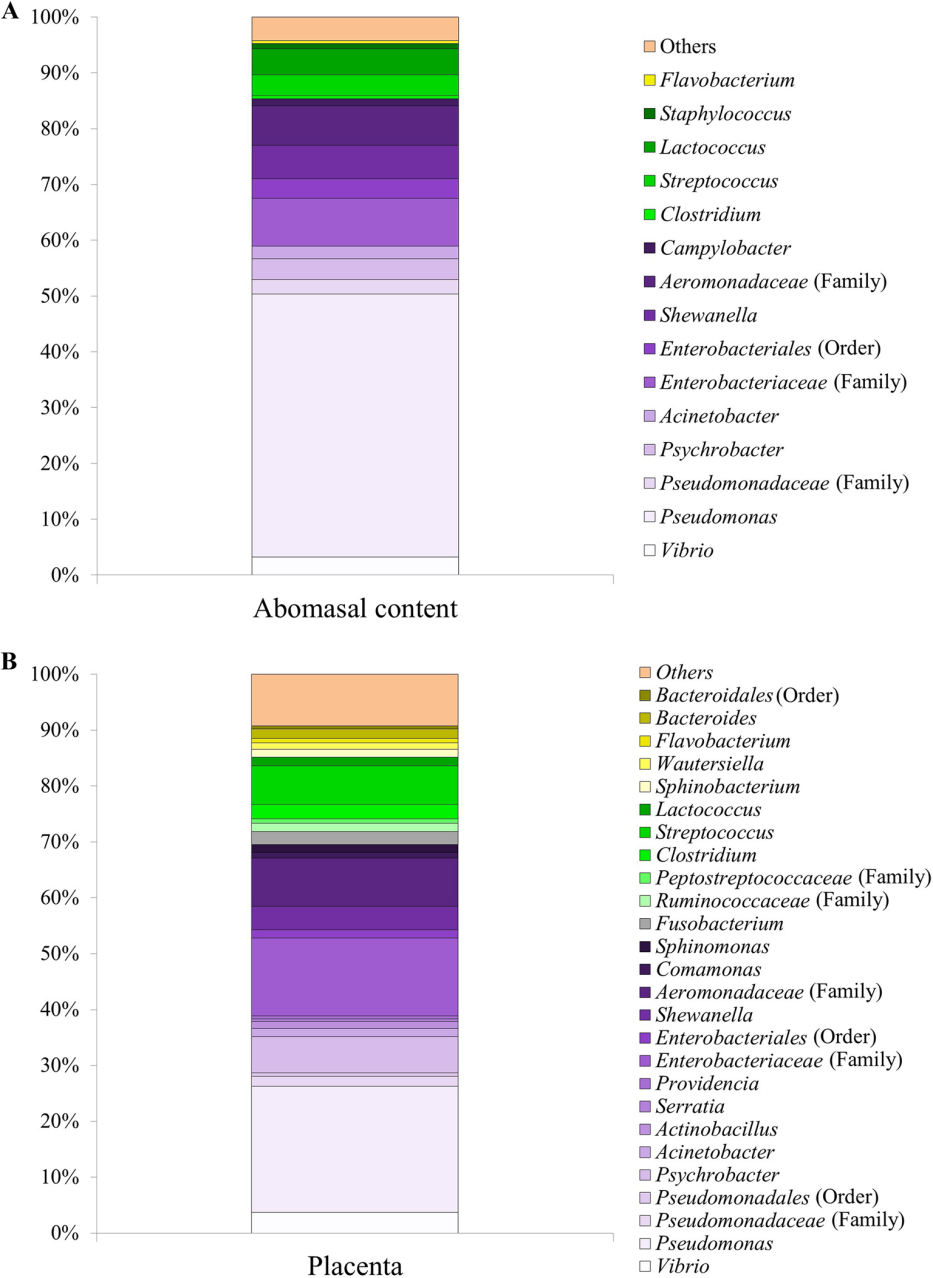
**Most abundant genera present in abomasal content (A) and placenta (B) (only taxa with relative abundances of** **≥** **0.5%).**

**Figure 5:**
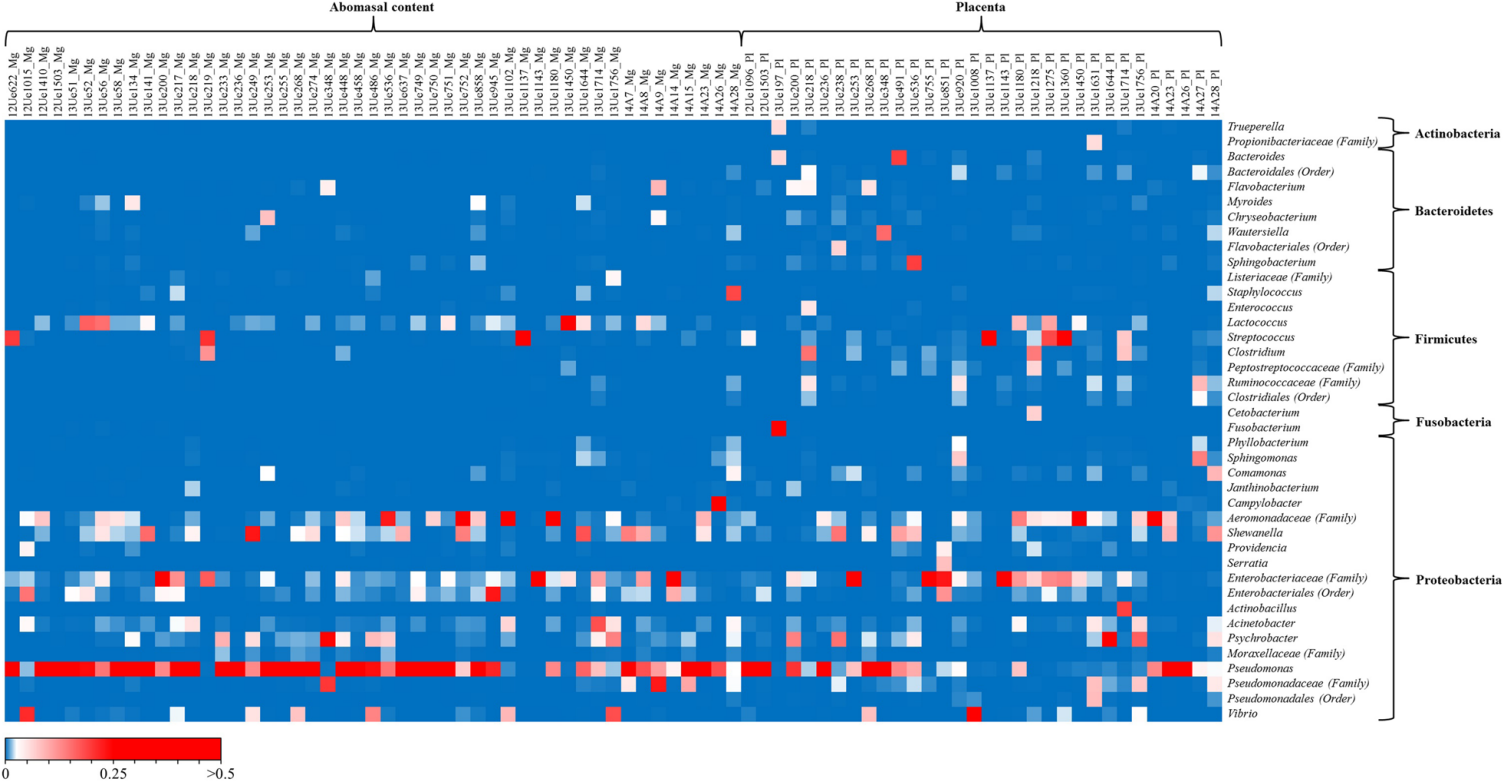
**Heat map showing the relative abundances of the most abundant genera identified in the AC and PL microbiota (only taxa with relative abundances of** **≥** **0.5%).**

At the genus level, 267 taxa were observed in the samples (AC = 162; PL = 255); however, 28.1% of the sequences could not be identified at the genus level. The most abundant genus was *Pseudomonas* (AC = 47.14%; PL = 22.56%), followed by unclassified genera derived from *Enterobacteriaceae* other (AC = 8.60%; PL = 13.92%) and *Aeromonadaceae* other (AC = 7.09%; PL = 8.60%) (Figures 4, 5).

### Culture

Only 8 of the 64 cases contained a possible abortive bacterial agent in large quantity and pure culture (Table 2): *Escherichia* (*E.*) *coli* (*n* = 2), *S. uberis* (*n* = 2), *Campylobacter* (*C.*) *fetus* subsp. *fetus* (*n* = 1), *Lactococcus* (*L*.) *lactis* (*n* = 1), *Trueperella* (*T.*) *pyogenes* (*n* = 1) and *Vibrio* (*V.*) *metschnikovii* (*n* = 1). No growth was observed for the healthy fetal samples on any culture media.

**Table 2:** **Abortive agents isolated in pure culture/large number and the corresponding most abundant genera found by NGS**

Sample ID	Organ	Abortive agents isolated in pure culture	Most abundant genera found by NGS (≤ 75% of the reads)
13Ue197_Pl	PL	*T. pyogenes*	*Fusobacterium* (74.04%), *Trueperella* (11.28%), others (14.71%)
13Ue755_Pl	PL	*E. coli*	*Enterobacteriaceae* ^a^ (94.2%), others (5.8%)
13Ue1008_Pl	PL	*V. metschnikovii*	*Vibrio* (89.92%), others (10.08%)
13Ue1137_Mg	AC	*S. uberis*	*Streptococcus* (99.62%), others (0.38%)
13Ue1137_Pl	PL	*S. uberis*	*Streptococcus* (99.33%), others (0.67%)
13Ue1143_Mg	AC	*E. coli*	*Enterobacteriaceae* ^a^ (99.17%), others (0.83%)
13Ue1143_Pl	PL	*E. coli*	*Enterobacteriaceae* ^a^ (96.18%), others (3.82%)
13Ue1275_Pl	PL	*S. uberis*	*Streptococcus* (37.24%), *Enterobacteriaceaea* (25.92%), *Lactococcus* (21.27%), others (15.57%)
13Ue1450_Mg	AC	*L. lactis*	*Lactococcus* (86.52%), others (13.48%)
14A26_Mg	AC	*C. fetus* subsp. *fetus*	*Campylobacter* (60.38%), *Pseudomonas* (36.03%), others (3.59%)
14A26_Pl	PL	*C. fetus* subsp. *fetus*	*Pseudomonas* (98.97%), others (1.03%)

### Histopathology

Histopathological results are summarized in Table 3. For two of the 17 cases evaluated, the NGS method revealed that more than 85% of the reads belonged to the genus *Pseudomonas*. One case (12Ue1096_Pl) revealed mild necrotizing placentitis with vasculitis and mixed inflammatory infiltrate characterized by neutrophils, lymphocytes and macrophages. The second case (12Uel503_Pl) displayed multifocal acute necrosis but without the associated inflammatory infiltrate.

**Table 3:** **Comparison of histopathological analysis and most abundant genera found by next generation sequencing (NGS) in available placenta samples**

Sample ID	Placentitis	Necrosis	Type of infiltrate	Vasculitis	Presence of ICB^a^	Presence of ECB^b^	Most abundant genera found by NGS (≤ 75% of the reads)	Comments
12Ue1096_Pl	Mild	Mild	Mixed	Yes	Yes	Yes	*Pseudomonas* (86.72%), others (13.28%)	
12Ue1503_Pl	No	Moderate	No	No	No	Yes	*Pseudomonas* (91.26%), others (8.74%)	*Pseudomonas* (99.68%) in the abomasal content
13Ue218_Pl	No	No	No	No	No	Yes	*Clostridium* (29.94%)*, Enterococcus* (9.71%), *Ruminococcaceae* ^a^ (9.67%), *Flavobacterium* (6.89%), *Bacteroidales* ^b^ (5.18%), *Enterobacteriaceae* (4.46%), *Pseudomonas* (3.32%), *Succinivibrio* (2.88%), RF16 (2.43%), others (22.63%)	
13Ue238_Pl	NA	NA	NA	NA	NA	NA	*Psychrobacter* (30.22%), *Shewanella* (29.58%), *Flavobacteriales* ^b^ (12.76%), *Pseudomonadaceae* ^a^ (4.50%), others (22.94%)	Severe autolysis
13Ue491_Pl	Mild	Moderate	Mononuclear	No	No	No	*Bacteroides* (39.09%), *Pseudomonas* (26%), *Shewanella* (21.69%), others (13.22%)	
13Ue851_Pl	Moderate	Moderate	Neutrophilic	Yes	No	No	*Enterobacteriaceae* ^a^ (46.46%), *Enterobacteriales* ^b^ (24.33%), *Serratia* (16.14%), others (13.07%)	
13Ue1008_Pl	Moderate	Moderate	Mixed	Yes	No	Yes	*Vibrio* (89.92%), others (10.08%)	*V. metschnikovii* isolated in culture
13Ue1137_Pl	Moderate	Moderate	Mixed	Yes	No	Yes	*Streptococcus* (99.33%), others (0.67%)	*Streptococcus* (99.62%) in the abomasal content, *S. uberis* isolated in culture
13Ue1143_Pl	Mild	Mild	Neutrophilic	No	No	Yes	*Enterobacteriaceae* ^a^ (96.19%), others (3.81%)	*Enterobacteriaceae* ^a^ (99.17%) in the abomasal content. *E. coli* isolated in culture
13Ue1218_Pl	Mild	Mild	Mixed	Yes	No	Yes	*Clostridium* (28.33%), *Cetobacterium* (13.16%), *Enterobacteriaceae* ^a^ (13.02%), *Peptostreptococcaceae* ^a^ (12.42%), *Aeromonadaceae* ^a^ (9.8%), others (23.27%)	High level of contamination with plants and feces
13Ue1275_Pl	Mild	Mild	Mixed	Yes	No	No	*Streptococcus* (37.24%), *Enterobacteriaceae* ^a^ (25.92%), *Lactococcus* (21.27%), others (15.57%)	*S. uberis* isolated in pure culture
13Ue1631_Pl	NA	NA	NA	NA	NA	NA	*Pseudomonadales* ^b^ (16.87%), *Pseudomonadaceae* ^a^ (16.24%), *Propionibacteriaceae* ^a^ (11.03%), *Acinetobacter* (9.13%), *Aeromonadaceae* ^a^ (6.81%), Candidatus *Portiera* (5.66%), *Ruminococcaceae* ^a^ (3.62%), *Psychrobacter* (3.33%), others (23.34%)	Severe autolysis
13Ue1644_Pl	No	No	No	No	No		*Psychrobacter* (83.99%), others (16.01%)	
13Ue1714_Pl	NA	NA	NA	NA	NA	NA	*Actinobacillus* (38.74%), *Clostridium* (15.78%), *Streptococcus* (14.54%), *Enterobacteriaceae* ^a^ (7.83%), others (23.11%)	Severe autolysis
13Ue1756_Pl	NA	NA	NA	NA	NA	NA	*Psychrobacter* (33.24%), *Pseudomonadaceae* ^a^ (15.06%), *Aeromonadaceae* ^a^ (13.06%), *Acinetobacter* (12.23%), *Vibrio* (5.53%), others (20.88%)	Severe autolysis
14A20_Pl	Mild	Mild	Mixed	No	No	Yes	*Aeromonadaceae* ^a^ (73%), *Pseudomonas* (26.26%), others (0.74%)	
14A28_Pl	NA	NA	NA	NA	NA	NA	*Shewanella* (24.04%), *Camomonas* (17.94%), *Psychrobacter* (9.97%), *Pseudomonadaceae* ^a^ (8.82%), *Pseudomonas* (4.49%), *Acinetobacter* (4.5%), *Wautersiella* (3.66%), *Staphylococcus* (3.64%), others (22.94%)	Severe autolysis

Cases with positive results for parasitology, virology and/or with presence of intralesional fungal organisms were not included.

^a^Presence of intracytoplasmic bacteria (ICB).

^b^Presence of extracellular bacteria (ECB).

Moderate necrotizing placentitis with mixed inflammatory infiltrate and vasculitis was present in a sample (13Ue1008_Pl) from which 89.92% of the reads belonged to the genus *Vibrio*. The corresponding bacterial culture yielded pure growth of *V. metschnikovii*.

Similarly, in cases with a high number of reads for *Streptococcus* that were identified as *S. uberis* in pure culture (13Ue1137_Pl and 13Ue1275_Pl), mild to moderate necrotizing placentitis with mixed inflammatory infiltrate and vasculitis was observed. Mild suppurative placentitis without vasculitis was the primary lesion in a case with 96.19% of reads belonging to the *Enterobacteriaceae* family. In this case, *E. coli* was isolated in pure culture (13Ue1143_Pl). The case (14A20_Pl) with a high number of reads for *Aeromonadaceae* (73%) displayed a mild necrotizing placentitis with mixed inflammatory infiltrate without vasculitis.

While the samples from 13Ue491_Pl, 13Ue851_Pl and 13Ue1218_Pl showed lesions as described below, we did not identify a specific possible pathogen from a high number of reads and/or isolated in culture. The sample 13Ue491_Pl showed a mild necrotizing placentitis with lymphohistiocytic inflammation. Moderate suppurative placentitis with necrosis and vasculitis was present in sample 13Ue851_Pl. In sample 13Ue1218_Pl, a mild necrotizing placentitis with mixed inflammatory infiltrate and vasculitis was observed.

Two samples did not show lesions. Five samples presented severe autolysis and were not suitable for identifying lesions.

**Figure 6:**
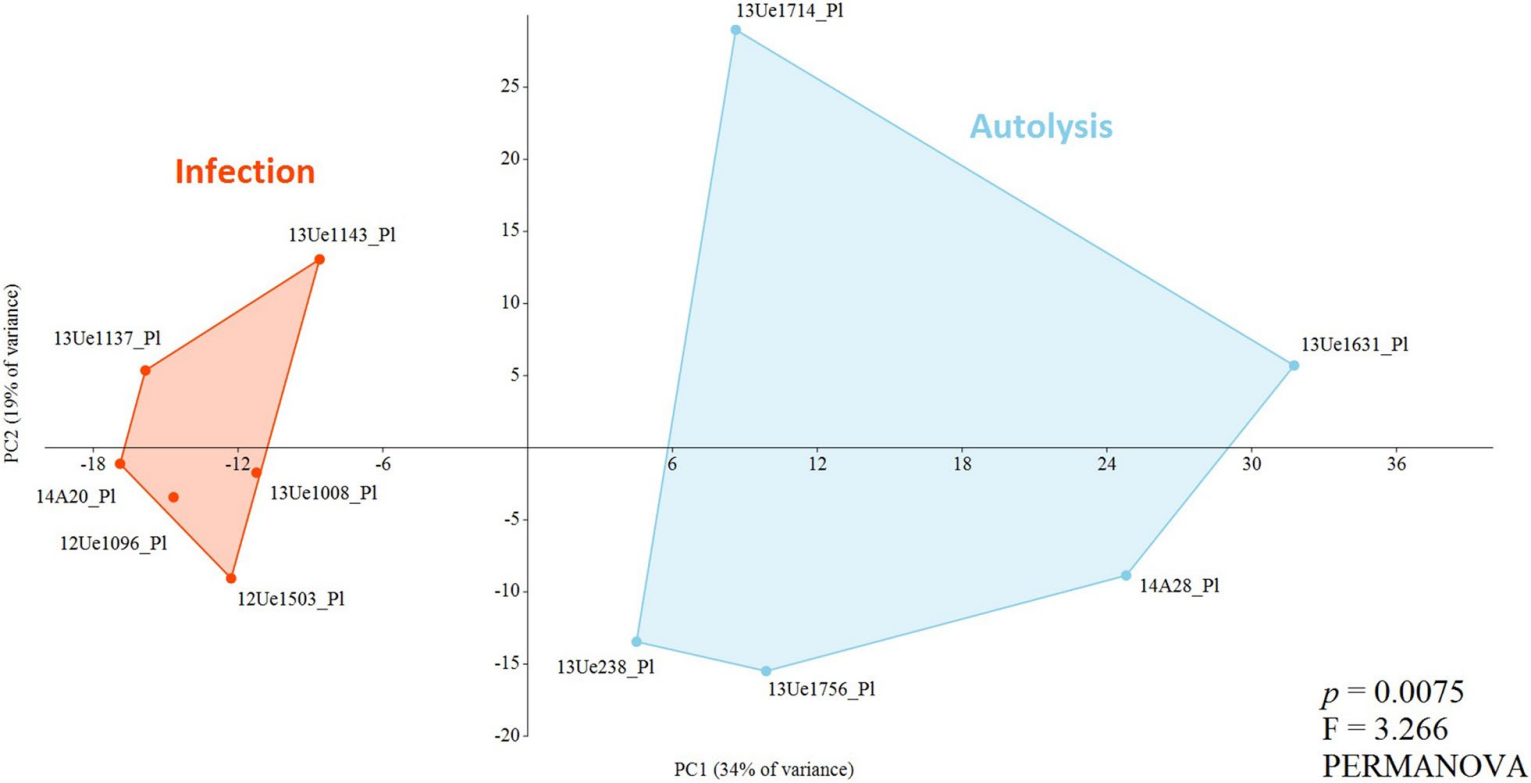
**Two-dimensional ordination of the microbial profiles of the placentas presenting infection or autolysis by principal component analysis (PCA).** Significant differences; *p* < 0.01, PERMANOVA.

Figure 6 shows the principal component analysis of the microbial profile for the placentas that presented lesions associated with an infection and the placentas presenting severe autolysis. Placentas presenting clustered lesions and showed less variability than the placentas with autolysis. Significant differences were observed between the two groups (*p* = 0.0075; F = 3.266; PERMANOVA).

## Discussion

Studies on the bovine microbiota have gained importance in the last 5 years with the characterization of the microbiota of the rumen [21–24], the complete gastrointestinal tract [25], the nasopharynx [26], milk [27–29], the teat [30] and the vagina and/or uterus [31–34]. In contrast to previous studies that focused on implicating specific bacteria from bovine abortion material by bacterial culture and targeted molecular detection [4, 6, 35–38], we studied the total bacterial microbiota present in abortion material from cattle using a 16S rRNA amplicon sequencing approach. In addition, we used a broad-spectrum bacterial culture to compare and confirm the sequencing results.

This study revealed that the taxonomic groups present in the bovine abortion material were Proteobacteria (AC = 87.35%; PL = 72.13%), Firmicutes (AC = 10.51%; PL = 15.66%) and Bacteroidetes (AC = 1.99%; PL = 7.81%). In a previous publication, the microbiota from the abomasal mucosa of healthy dairy cattle exhibited relative abundances of Firmicutes (27.4%), Bacteroidetes (20.95%) and Proteobacteria (19.82%) [25]. The composition profiles from the placenta and abomasal content showed significant differences (*p* = 0.0001), with the placenta exhibiting more taxa, while some phyla were not present in the abomasal content. This is not surprising since the placenta is often contaminated with bacteria from the vagina and vulva as well as the environment [7, 39]. Therefore, the infectious process of specific abortigenic bacteria, such as *Chlamydia* spp., may be confined to the placenta, which makes this organ indispensable to thoroughly investigate the abortion material [39, 40].

Figure 5 shows that the microbiota varied between the different abortion cases pointing at a possible causative genus. Some cases showed one prevalent taxonomic unit (one red square present) with many reads associated with lesions in the placenta, whereas other cases had profiles that were more equally distributed (presenting several squares from light blue to red) and were devoid of histological lesions. After interpretation of results, it was possible to reveal different taxonomic profiles showing two principal components accounting for 53% of the total data variability and thereby clearly splitting autolytic samples from those with infectious bacterial causes on the X-axis (Figure 6). These observations followed the key points for establishing an etiological diagnosis and distinguishing the exogenous microbiota from a real fetal infection [40, 41]. These points include isolating the agent in a relatively pure culture and/or a large number from the fetal tissues and/or the placenta and the presence of gross and microscopic lesions consistent with a bacterial infection in the placenta. Ideally, additional tests should exclude other common abortigenic agents including viruses, fungi and parasites. Our findings revealed opportunistic abortive agents that are not detected by the routine diagnostic approach that focuses on epizootic bacterial agents. Most of the bacterial agents that cause abortion in cattle are commensal or environmental bacteria that, as opportunistic pathogens, produce sporadic abortions [5]. These opportunistic organisms are usually categorized into two groups: (1) bacteria belonging to the commensal microbiota of the mucosal surfaces and (2) common environmental bacteria [39]. Most reports, including the broad-spectrum screening of bovine abortions, are based on bacterial cultures [42–44]. Culturing may be a powerful method for isolating bacteria if the conditions are compatible with the microbial target and sample type, but it may fail to identify novel or unculturable pathogens [45, 46]. As expected, the NGS method revealed many genera that were not evident from bacterial cultures. One of the advantages of the parallel culture-based approach is the ability to assign species-level identification and overcome the restricted taxonomic result of the NGS method that amplifies only a fragment of the 16S rRNA gene [47]. The following opportunists were identified by NGS and isolated in pure culture (Table 2): *Vibrio* (identified as *V. metschnikovii* in culture), *Streptococcus* (identified as *S. uberis* in culture), *Lactococcus* (identified as *L. lactis* in culture), *Trueperella* (identified as *T. pyogenes* in culture), unidentified *Enterobacteriaceae* (identified as *E. coli* in culture), and *Campylobacter* (identified as *C. fetus* subsp. *fetus* in selective culture). For *V. metschnikovii*, *S. uberis*, *L. lactis* and *E. coli*, tissue was available for histopathological analysis, and we confirmed lesions in the placentas. In the cases with placental lesions in which a possible agent was only detected by NGS with a high number (≥70%) of reads, we detected unidentified *Pseudomonas* and unidentified *Aeromonadaceae*.

*Vibrio metschnikovii* has been identified by bacteriological culture in cattle, swine and horse abortions [48], and *L. lactis*, which is mainly associated with bovine mastitis [49], has also been isolated from a bovine abortion [43]. It is likely that the suspected abortive agents, unidentified *Pseudomonas*, *S. uberis* and *E. coli*, may spread via the amniotic-oral route to the fetus and produce infection (Table 3); however, causality between these pathogens and abortions has not been confirmed [42, 50, 51]. Interestingly, *Pseudomonas* was the most prevalent genus revealed by the NGS approach and it was present in all the samples analyzed (Figure 5). The implication of *Pseudomonadaceae* in human periodontitis was previously revealed by NGS [52]. A previously unidentified member of the family *Aeromonadaceae*, which was producing placental lesions, was later identified by NGS. This suggests that culturing opportunistic environmental bacteria may be difficult. To our knowledge, only two previous studies, reported in 1972 [53] and 1993 [51], identified *Aeromonas hydrophila* and *Aeromonas* spp. as etiological agents of bovine abortion.

One pure culture of *T. pyogenes* partially matched the NGS results since the number of reads was lower (11.28%) than those of the most abundant genus, *Fusobacterium* (74.04%). *T. pyogenes* cooperates with environmental anaerobes, such as *Fusobacterium necrophorum*, to produce infections, such as endometritis in cows, and increases the possibility of uterine inflammatory conditions while intensifying disease symptoms [54–56]. *Fusobacterium* is known to cause sporadic bovine abortion [57, 58], but the impact of mixed infections on abortions has not yet been determined. Moreover, anaerobic bacteria may be underestimated as abortifacients since anaerobic cultures are not normally part of routine diagnostic procedures [41]. The NGS approach was also able to identify multiple possible abortive taxa from the same sample in 13Ue851_Pl and 13Ue1275_Pl (Table 3). Unidentified *Enterobacteriaceae* (46.46%) were found together with *Enterobacteriales* (24.33%) and *Serratia* (16.14%), and *S. uberis* (37.24%) were found alongside *Enterobacteriaceae* (25.92%) and *Lactococcus* (21.27%). As previously suggested, mixed infections may play an underestimated role in ruminant abortions [4].

It is often difficult to assess the presence of potentially infectious bacteria in cultures, particularly in cases where rare opportunistic agents, e.g., *Lactococcus*, *Streptococcus* or *Vibrio*, are identified, as they tend to be overlooked as contaminants. The amplicon sequencing approach however, gives additional information through the number of reads obtained [59]. This is especially noteworthy in the case of *Pseudomonas* that was not apparent in the culture. Another advantage is the ability to simultaneously screen for the broadest possible bacterial variety without requiring a costly detection method, such as qPCR, DNA arrays or antibody detection [59]. Interpretation of the NGS results must be done cautiously though, keeping in mind that further analyses are needed to determine causality. Another challenge in investigating cattle abortion is that the sample site may not correspond with the location of the active infection [60]. Additionally, depending on the DNA extraction method and the amplified variable regions of the 16S rRNA, some bacteria may be over- or underrepresented [61, 62]; e.g., we did not detect *Chlamydia*, *Leptospira* or *Coxiella* in the sequencing results, whereas these bacteria have been reported in bovine abortions in Switzerland [4]. This might be due to a lower affinity of the primers or a lower taxonomic resolution of the amplified fragment to the specific 16S rRNA gene in these pathogens. These limitations of the amplicon sequencing approach have previously been discussed in view of its suitability for diagnostics [45].

Histopathological analysis of tissue with a high degree of autolysis is not recommended due to loss of tissue architecture and cellular detail. NGS, however, allowed us to associate a specific microbiota to the autolytic tissue. Unlike the infectious cases, the autolytic placentas did not show a dominant taxon. In these cases, a variety of bacterial genera, represented at a similar percentage of reads, appeared to be involved in the tissue spoilage (Table 3, Figure 6). Most of these bacterial genera are also causative agents of meat spoilage [63, 64]. Moreover, a high grade of contamination with commensal microbiota from the gastrointestinal tract, such as the families *Rickenellaceae*, *Ruminococcaceae*, *Peptostreptococacceae*, *Enterobacteriaceae* or the order *Clostridiales* [25] was detected in some samples. The presence of other environmental or/and commensal bacteria can lower the ability to accurately detect an etiological agent as has been described for the parasite *Tritrichomonas foetus* [65]. Our results highlight the importance of adequately preserving the samples in sterile, chilled containers and rapidly transporting them to the diagnostic laboratory to avoid autolysis and contamination.

Although amplicon sequencing has been discussed as a diagnostic approach [60], the aim of our study was not the evaluation of NGS as a diagnostic tool but as a novel research approach to gain deeper insight into the microbiota present in abortion material. Our study emphasizes the difficulties of applying 16S amplicon sequencing to abortion diagnostics, such as the nature and suitability of the sample and the presence of contaminants. NGS helped us uncover abortifacients that went undetected by traditional methods and identify possible multi-infections. Nonetheless, standardization of workflows and cut-offs for diagnostic purposes based on the interpretation of percentage of reads is difficult to achieve and analysis would not be cost-efficient, especially since identification at species level cannot be achieved by NGS only but is required for an etiological diagnosis.

The targeted fragment of the 16S rRNA gene was not amplified in the three negative controls, indicating the absence of bacterial DNA; furthermore, no growth on the different culture media was observed. In our study, the negative control samples were extracted in the necropsy hall in aseptic conditions directly from the uterus of a dead cow having a minimal contact with the environment and no contact with the commensal microbiota from the vagina. Our ideal negative control sample would be a healthy fetus and the placenta extracted through the vaginal tract from an interrupted pregnancy of a healthy cow. However, from an animal welfare point of view this experimental setup would not be commensurate to the expected gain of knowledge and thus is not approved by the Swiss Federal Animal Protection Law (455, article 19, paragraph 4). A recent study reported the existence of a microbiota associated to the bovine placenta, but the authors could not exclude a possible contamination of the biopsies during sampling [66]. Moreover, the extraction of the mentioned biopsies was done through the vaginal tract of the live cows exposing the samples to the commensal microbiota. Although the authors took care to apply antimicrobial solution before and between sampling, this does not ensure elimination of DNA which then may persist, e.g. leading to the reported presence of the phyla Planctomycetes, a slow-growing decomposer of organic matter [67] and Euyarchaeota, a methanogenic archaea group from the rumen of cattle [68] in the placenta and the amniotic fluid. A previous work showed for the first time the presence of prenatal microbiota in human placentas [69]. However, a subsequent study concluded with a new sample set that the previous one could not distinguish between placental microbiota and contamination introduced during DNA purification [70]. Our findings indicate that under timely and sterile sampling conditions, bacterial microbiota is likely absent from these tissues.

Our study underlines the potential of amplicon sequencing to identify or confirm unknown etiological agents, such as unidentified *Pseudomonas*, *S. uberis*, *L. lactis, V. metschnikovii* and unidentified *Aeromonadaceae.* These new insights encourage adaptation of the diagnostic focus and extension of the spectrum of understudied opportunistic abortive bacteria.
